# Biosynthesis and Immobilization of Biofunctional Allophycocyanin

**DOI:** 10.1155/2011/751452

**Published:** 2011-08-10

**Authors:** Yingjie Chen, Shaofang Liu, Yulin Cui, Peng Jiang, Huaxin Chen, Fuchao Li, Song Qin

**Affiliations:** ^1^Key Laboratory of Experimental Marine Biology, Institute of Oceanology, Chinese Academy of Sciences, Qingdao 266071, China; ^2^Graduate University of Chinese Academy of Sciences, Beijing 100049, China

## Abstract

The holo-allophycocyanin-**α** subunit, which has various reported pharmacological uses, was biosynthesized with both Strep-II-tag and His-tag at the N-terminal in *Escherichia coli*. The streptavidin-binding ability resulting from the Strep II-tag was confirmed by Western blot. Additionally, the metal-chelating ability deriving from the His-tag not only facilitated its purification by immobilized metal-ion affinity chromatography but also promoted its immobilization on Zn (II)-decorated silica-coated magnetic nanoparticles. The holo-allophycocyanin-**α** subunit with streptavidin-binding ability was thereby immobilized on magnetic nanoparticles. Magnetic nanoparticles are promising as drug delivery vehicles for targeting and locating at tumors. Thus, based on genetic engineering and nanotechnology, we provide a potential strategy to facilitate the biomodification and targeted delivery of pharmacological proteins.

## 1. Introduction

Allophycocyanin (APC) is a biliprotein located in the core of the phycobilisome found in blue-green and red algae [1–4]. This biliprotein is composed of two different subunits, *α* and *β*, each subunit having one phycobilin (PCB; [5, 6]). As a bioactive protein, the pharmaceutical properties of APC have been demonstrated [7]. Primarily, the chromophore, a potent peroxyl radical scavenger, was thought to be mainly responsible for its antitumor activity [8, 9]. However, recent studies on the precise activity of apoprotein confirmed its important role in the bioactivities of APC [10, 11]. Through the development of recombinant DNA technology, holo-allophycocyanin subunits have been engineered and produced in large quantities [12, 13]. These subunits have shown even stronger radical scavenging abilities than apoprotein and native APC [13], suggesting the feasibility of biosynthesis for producing medical proteins. 

During the genetic engineering process, a number of antibody epitope tags have been exploited to confer target identification capability to recombinant proteins. Recently, Strep-II-tag, a short peptide sequence that provides proteins with streptavidin-binding ability, has received increasing attention with respect to the possibility that it might avoid chemical manipulation for the conjugation of antibodies to bioactive proteins [14–16]. In addition, a short His-tag, which is the most commonly used tag, can be easily fused to proteins without impairing their function and confer metal chelating ability [17]. His-tagged proteins can thus be readily purified in a single step by immobilized metal-ion affinity chromatography (IMAC; [18–20]). In addition, there is potential to specifically immobilize His-tagged proteins on a solid surface modified with metal cations [21–23]. Therefore, recombinant DNA technology has made it feasible to engineer and produce pharmaceutical proteins with bioaffinity and target identification capabilities.

Meanwhile, modern development of nanotechnology has produced novel carriers for the targeted delivery of drugs [24, 25], especially magnetic nanoparticles, which can be manipulated simply with an external magnetic field [25]. In these magnetic drug-delivery platforms, a silica coating is always used to reduce random fluctuations and biodegradations of the magnetic core, partly because of its heat resistance and biomolecule-binding abilities. Furthermore, exploitation of new materials has led to the creation of silica-coated magnetic nanoparticles modified with metal cations [26–29]. Thus, it is possible for magnetic nanoparticles to be utilized in the immobilization and delivery of His-tagged proteins.

In this work, based on both genetic engineering and nanotechnology, we developed a potential strategy to facilitate the biomodification and targeted delivery of pharmacological proteins. Zn (II)-decorated silica-coated magnetic nanoparticles (ZnSiMNPs) were prepared and characterized as protein carriers, while cyanobacterial allophycocyanin (APC), a unique and inexpensive pigment protein with various pharmacological uses, was chosen as a model protein. This biliprotein was biosynthesized with both His-tag and Strep-II-tag at the N-terminal in *Escherichia coli*. Finally, the protein was immobilized via the His-tag on ZnSiMNPs and its streptavidin-binding ability was confirmed. 

## 2. Materials and Methods

### 2.1. Construction of Expression Vectors

**Figure 1:**
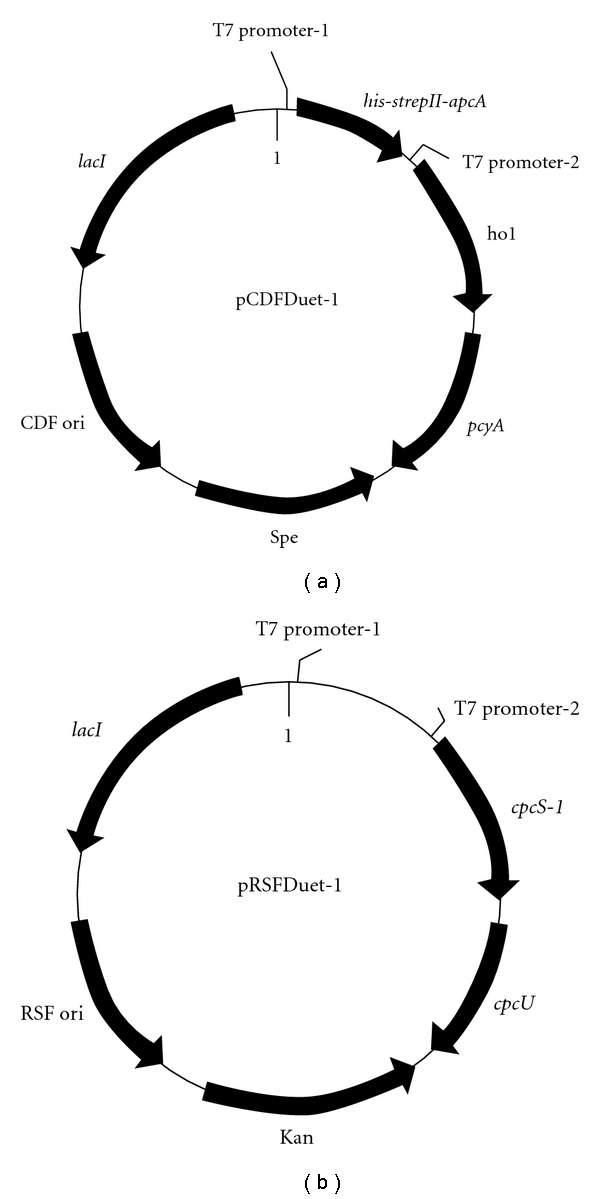
The pCDFDuet*-his-strepII-apcA-ho1-pcyA* and pRSFDuet*-cpcS-cpcU* expression vectors.

In general, standard procedures were used for DNA manipulation. All genes for biosynthesis of holo-APC-*α* subunit were PCR-amplified from *Synechocystis* sp. PCC6803 with specific primers. The primers used to amplify the *strepII-apcA *gene were 5′AAGGATCCGAGTAACTGGTCACACCCACAATTCGAGAAAATGAGTATCGTCACGAA3′ and 5′GCGAGCTCCTAGCTCATTTTTCCGAT3′. The other primers were similar to those described previously [3]. As shown by the schemes in Figure 1, the s*trepII-apcA *gene was fused to a His-tag and denoted as *his-strepII-apcA* for apo-APC-*α* with a His-Strep-II-tag at the N-terminal. The *ho1* and *pcyA* genes for the biosynthesis of PCB were ligated into the pCDFDeut-1 vector (Novagen), and the *cpcS *and *cpcU* genes for PCB attachment to apoproteins were ligated into the pRSFDeut-1 vector (Novagen). The final plasmid constructs were sequenced to check their veracity. 

### 2.2. Expression and Purification of the Recombinant Proteins

The pCDFDuet-*his-strepII-apcA-ho1-pcyA* and pRSFDeut*-cpcS-cpcU* expression vectors were cotransformed into* Escherichia coli* BL21. The transformants were selected by 50 *μ*g/mL spectinomycin and 50 *μ*g/mL kanamycin. A single colony of the transformed *Escherichia coli *BL21 was cultured in 5 mL of LB medium at 37°C overnight. The bacterial culture was then transferred into 400 mL of LB and cultured at 37°C. When the OD_600_ reached 0.6~0.8, the culture was induced with 0.5 mM isopropyl **β**-D-thiogalactoside at 28°C. Bacteria were harvested after 8 h induction and stored at −20°C before use.

To obtain the recombinant proteins, ultrasonicated bacteria supernatant was passed through a Ni^2+^-NTA affinity column (GE Healthcare Bio-Sciences). The retained protein on the column was eluted by 50 mM sodium phosphate and 300 mM imidazole, pH 7.4. The pooled elution was loaded onto a Sephadex G-25 size-exclusion column to eliminate imidazole. The protein fraction was collected and concentrated and analyzed by UV-visible and fluorescence spectroscopy.

### 2.3. Western Blotting Analysis

Western hybridization was performed with streptavidin-derivatized horseradish peroxidase to detect the bioaffinity of the holo-APC-*α* with tags. A molecular weight marker, a whole bacterial culture extract, and the protein solution purified above were run side by side on the gel in duplicate. After running, the gel was cut in half. One half was stained with Coomassie brilliant blue and the other was transferred to nitrocellulose following the kit manufacturer's instructions. This nitrocellulose was then incubated in TBS (25 mM Tris-HCl, 138 mM NaCl, and 2.68 mM KCl, pH 7.4) plus 0.05% polyoxyethylenesorbitan monolaurate (Tween 20) and 3% BSA for 1 h at room temperature. After a brief rinse with the same buffer without BSA, the blot was incubated with a streptavidin-derivatized horseradish peroxidase conjugate in TBS/0.05% Tween-20/3% BSA for 1 h at room temperature. It was then washed three times with TBS/0.05% Tween 20 and once with TBS. Finally, the HRP substrate was added and the blot was developed for approximately 3 min.

### 2.4. Preparation and Characterization of ZnSiMNPs

Silica-coated magnetic nanoparticles (SiMNPs) were first prepared with a water-in-oil microemulsion method described previously [30, 31]. Then, 1.0 wt.% SiMNPs were suspended in 5.0 mM ZnSO_4_ solution. After 10 min ultrasonic homogenization, the suspension was left to stir overnight at 25°C. The magnetic nanoparticles (ZnSiMNPs) were magnetically harvested and washed several times. The ZnSiMNPs were then suspended in water prior to use. The morphology of the nanoparticles was analyzed by transmission electron microscopy (TEM).

### 2.5. Immobilization and Imaging of Holo-APC-*α* on Magnetic Nanoparticles

The ZnSiMNPs or SiMNPs were added to a 0.5 mL holo-APC-*α* solution prepared as described above. The mixture was gently agitated at room temperature for 30 min. Nanoparticles were separated from the remaining solution with a magnetic rack. The nanoparticles were washed and suspended in 50 mM sodium phosphate, pH 7.4. In order to test the retentive bioaffinity of proteins loaded onto the nanoparticles, 10 *μ*L of 1 mg/mL streptavidin-FITC was added to the suspension. After incubation for 30 min, the loaded nanoparticles were collected, washed, and suspended in 50 mM sodium phosphate, pH 7.4, then analyzed with fluorescence microscopy.

## 3. Results

### 3.1. Bioaffinity of Recombinant Protein

**Figure 2:**
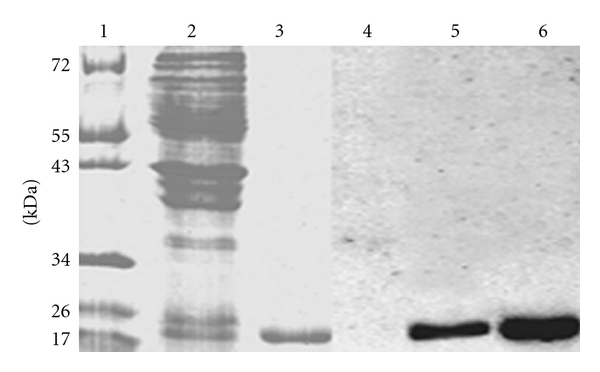
SDS-PAGE analyses indicate the expression of His-Strep-II-holo-APC-*α* and its bioaffinity. Lanes from 1 to 3 were stained by Coomassie brilliant blue: 1: marker; 2: whole protein in bacteria extract; 3: purified protein. Lanes 4 to 6 show the results for western blotting: 4: marker; 5: whole protein in bacteria extract; 6: purified protein.

After 8 h induction, the color of the bacteria in the fermentation solution changed to cyan. The cells were collected and ultrasonically disrupted, and the cyanosupernatant was purified with a Ni^2+^-NTA affinity column. The eluted solution from this column was clearly blue. Results of SDS-PAGE and Western blotting analyses for the whole bacteria cell extract and purified proteins are shown in Figure 2, showing many protein bands in the whole bacteria extract. A single-protein-band protein near 22 kDa, corresponding to the calculated molecular masses of holo-APC-*α* with His-tag and Strep-II-tag, resulted from the protein sample purified by the Ni^2+^-NTA affinity column. The selective purification of this recombinant protein by the Ni^2+^-NTA affinity column confirmed the inclusion of an active His-tag. Western blot analysis showed a single band at a similar location to the SDS-PAGE results for the whole bacterial cell extract and purified protein, indicating the presence of the Strep-II-tag and confirming its bioaffinity. 

### 3.2. Spectra of Recombinant Protein

**Figure 3:**
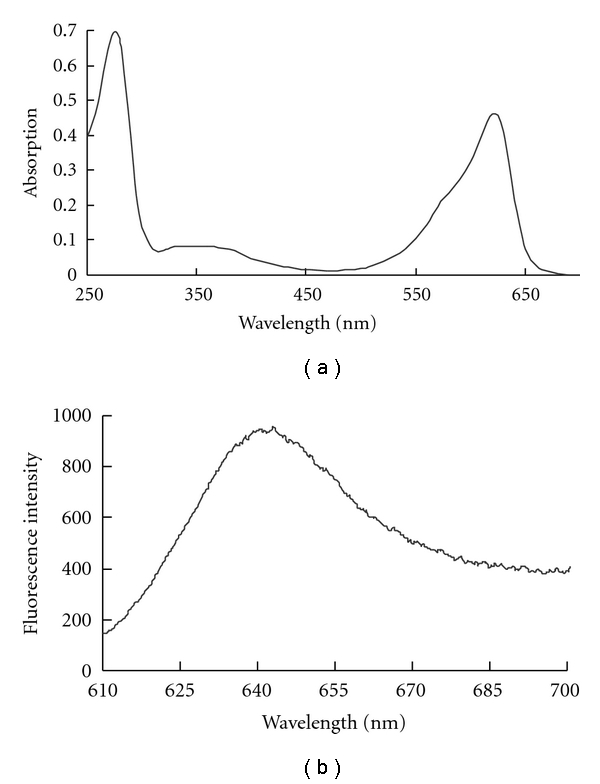
UV-vis absorption (a) and fluorescence emission spectra (b) for the purified His-Strep-II-holo-APC-*α* indicate the correct attachment of PCB on the apoprotein.

The purified protein solution had an adsorption maximum at 615 nm and a fluorescence emission maximum at 643 nm (Figure 3). The absorption and fluorescence spectra of the holo-APC-*α* recombinant with His-tag and Strep-II-tag were consistent with those reported for the native protein [12], indicating the correct attachment of PCB on the apoprotein.

### 3.3. Characterization of ZnSiMNPs

**Figure 4:**
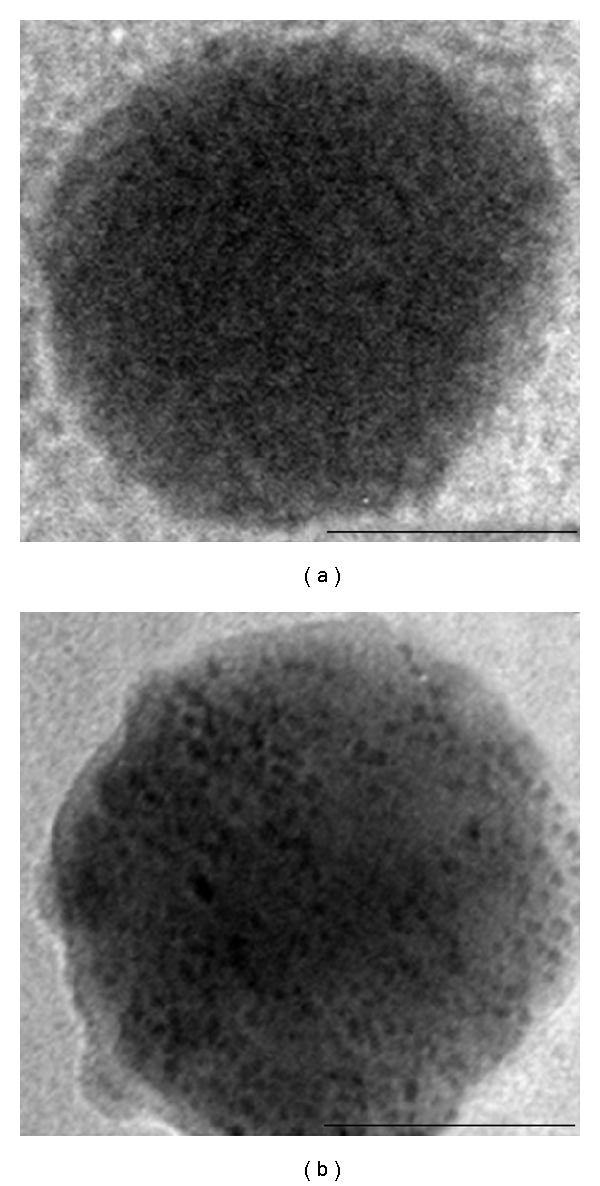
TEM images of the smooth surface of SiMNPs (a) and the semishell of ZnSiMNPs (b). Bars: 50 nm.

TEM images of the SiMNPs and ZnSiMNPs are shown in Figure 4. These two types of spherical nanoparticles had a similar size, close to 100 nm. However, the surface of SiMNPs appeared somewhat smooth, while ZnSiMNPs were evidently decorated with 3–5 nm nanoparticles, which had the appearance of a semishell. This semishell appearance was similar to the decoration of nanophasic nickel on silica microspheres [27], so it is likely that the coating was caused by the deposition of zinc on silica.

### 3.4. Immobilization and Bioaffinity of Protein on ZnSiMNPs

**Figure 5:**
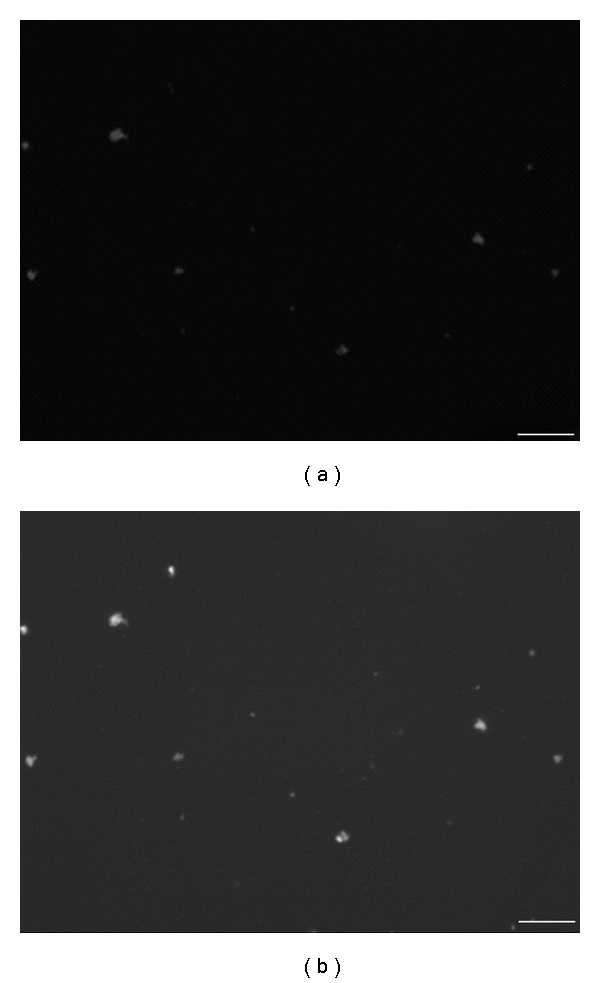
Fluorescence microscopy images of His-Strep-II-holo-APC-*α* laden ZnSiMNPs after hybridization with streptavidin-FITC: excited by green (a) or blue (b) light, confirming the surface immobilization of holo-APC-*α* and the retentive streptavidin-binding ability of proteins loaded onto the nanoparticles. Bars: 1 *μ*m.

After incubation with His-Strep-II-holo-APC-*α*, nanoparticles were separated with a magnet. ZnSiMNPs changed color from brown to blue, while the SiMNPs remained brown. This visible difference indicates the key role of zinc decoration on the surface immobilization of His-Strep-II-holo-APC-*α* on the nanoparticles. In order to further verify the bioaffinity of the protein immobilized on the ZnSiMNPs, streptavidin-FITC was used. As shown in Figure 5, ZnSiMNPs showed a fluorescent signal, representative of holo-APC-*α* or FITC with the excitation of green or blue light, respectively, suggesting the surface immobilization of holo-APC-*α* and the retentive streptavidin-binding ability of proteins loaded onto the nanoparticles. As a control, SiMNPs showed no fluorescence upon excitation by either green or blue light.

## 4. Discussion

**Figure 6:**
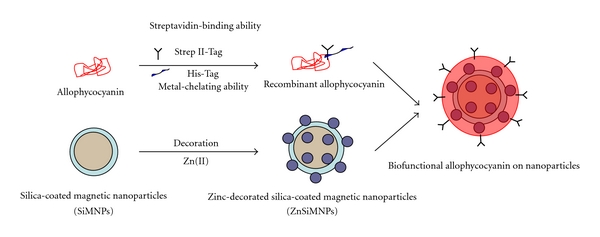
Schemes for potential strategies to facilitate biomodification of nanoparticles and targeted delivery of pharmacological proteins.

Based on genetic engineering and nanotechnology, this study demonstrated a potential strategy to facilitate the biomodification and targeted delivery of pharmacological proteins. This strategy is represented schematically in Figure 6. Cyanobacterial allophycocyanin, a unique and inexpensive pigment protein with various pharmacological uses, was chosen as a model protein. This research reports, for the first time, the recombinant construction of holo-APC-*α* with both a His-tag and a Strep-II-tag. This recombinant protein had similar spectral properties to native holo-APC-*α* and showed streptavidin-binding capability due to the fusion of the Strep-II-tag. In addition, the metal-chelating ability derived from the His-tag not only facilitated its purification but also promoted its immobilization on ZnSiMNPs. Together with the results of electrophoresis, Western blotting, and spectral analysis, we concluded that holo-APC-*α* engineered with a His-tag and a Strep-II-tag was successfully biosynthesized. This recombinant protein could be immobilized on ZnSiMNPs and modified with streptavidin without chemical manipulation. Furthermore, the utilization of magnetic nanoparticles as drug delivery carriers is promising for targeting delivery to tumor sites.
